# CRISPR-Cas9-based approaches for genetic analysis and epistatic interaction studies in *Coxiella burnetii*

**DOI:** 10.1128/msphere.00523-24

**Published:** 2024-11-19

**Authors:** Samuel Steiner, Craig R. Roy

**Affiliations:** 1 Department of Microbial Pathogenesis, Yale University School of Medicine, New Haven, Connecticut, USA; University of Kentucky College of Medicine, Lexington, Kentucky, USA

**Keywords:** *Coxiella burnetii*, base editing, CRISPR, CRISPRi, intracellular infection

## Abstract

*Coxiella burnetii* is an obligate intracellular bacterial pathogen that replicates to high numbers in an acidified lysosome-derived vacuole. Intracellular replication requires the Dot/Icm type IVB secretion system, which translocates over 100 different effector proteins into the host cell. Screens employing random transposon mutagenesis have identified several *C. burnetii* effectors that play an important role in intracellular replication; however, the difficulty in conducting directed mutagenesis has been a barrier to the systematic analysis of effector mutants and to the construction of double mutants to assess epistatic interactions between effectors. Here, two CRISPR-Cas9 technology-based approaches were developed to study *C. burnetii* phenotypes resulting from targeted gene disruptions. CRISPRi was used to silence gene expression and demonstrated that silencing of effectors or Dot/Icm system components resulted in phenotypes similar to those of transposon insertion mutants. A CRISPR-Cas9-mediated cytosine base editing protocol was developed to generate targeted loss-of-function mutants through the introduction of premature stop codons into *C. burnetii* genes. Cytosine base editing successfully generated double mutants in a single step. A double mutant deficient in both *cig57* and *cig2* had a robust and additive intracellular replication defect when compared to either single mutant, which is consistent with Cig57 and Cig2 functioning in independent pathways that both contribute to a vacuole that supports *C. burnetii* replication. Thus, CRISPR-Cas9-based technologies expand the genetic toolbox for *C. burnetii* and will facilitate genetic studies aimed at investigating the mechanisms this pathogen uses to replicate inside host cells.

**IMPORTANCE:**

Understanding the genetic mechanisms that enable *C. burnetii* to replicate in mammalian host cells has been hampered by the difficulty in making directed mutations. Here, a reliable and efficient system for generating targeted loss-of-function mutations in *C. burnetii* using a CRISPR-Cas9-assisted base editing approach is described. This technology was applied to make double mutants in *C. burnetii* that enabled the genetic analysis of two genes that play independent roles in promoting the formation of vacuoles that support intracellular replication. This advance will accelerate the discovery of mechanisms important for *C. burnetii* host infection and disease.

## INTRODUCTION

*Coxiella burnetii*, the causative agent of the zoonotic disease Q fever, is an obligate intracellular pathogen of mammals that employs an understudied infection strategy by replicating in an acidified lysosome-derived organelle, an environment hostile to most microbes (1–8). Thus, *C. burnetii* has emerged as an important model organism for the investigation of the molecular and cellular processes underlying host–pathogen interactions and the subversion of host cell functions (9–11). Upon entry into mammalian cells, the bacteria remain in a membrane-bound compartment and are transported through the endocytic pathway that promotes fusion of the phagosome with lysosomes. Upon delivery to an acidified lysosome-derived compartment, the bacteria become metabolically active and orchestrate the biogenesis of a spacious organelle called the *Coxiella*-containing vacuole (CCV) that is permissive for bacterial replication (1, 12, 13). Individual CCVs in a cell formed by *C. burnetii* that were internalized independently undergo homotypic fusion by a process that requires the host autophagy system, thus giving rise to a single mature CCV that expands during bacterial replication and will occupy most of the host cell cytoplasm (14). This unique, highly fusogenic organelle has autolysosomal characteristics and is positive for proteins that localize to late endosomes, lysosomes, and autophagosomes, including Rab7, V-ATPase, VAMP7, LAMP-1, Cathepsin D, p62, and LC3 (14–22). The process of CCV biogenesis and maturation is driven by the collective activity of an arsenal of over 100 effector proteins, which are translocated into the host cell cytosol by the bacterial Dot/Icm type IVB secretion system (23, 24). This multiprotein nanomachine functions analogously to the Dot/Icm system of the evolutionarily related pathogen *Legionella pneumophila* and is assembled and activated as *C. burnetii* becomes metabolically active upon acidification of the CCV (25–33). Although there has been extensive research on how individual Dot/Icm effectors subvert eukaryotic host cell pathways to promote CCV maturation and bacterial replication, the cellular targets, biochemical activities, and molecular functions of most *C. burnetii* effectors are unknown (34, 35).

The proteins Cig57 and Cig2 (CvpB) are two effectors with demonstrated important roles for CCV biogenesis and intracellular replication (14, 36). Cig57 is involved in recruiting clathrin to the CCV by interacting with FCHO2, an accessory protein for clathrin-mediated endocytosis (37). A *cig57* mutant has a pronounced intracellular replication defect (14, 37). Cig2 is required for fusion of the CCV with autophagosomes and is involved in promoting homotypic fusion of individual CCVs within infected cells. A *C. burnetii* mutant deficient in *cig2* displays a multivacuole phenotype (14, 15, 36).

Recent advances in axenic cultivation, transformation, and genetic manipulation of *C. burnetii* have facilitated the identification and characterization of virulence factors (38–42). Apart from using random transposon mutagenesis to generate gene disruption mutants, strategies to create targeted gene deletion mutants in *C. burnetii* have been described. These include a Cre-*lox*-driven recombination system and a loop-in/loop-out allelic exchange procedure dependent on a target template encoded on a suicide plasmid (43, 44). Although several genes have been deleted using one of these site-directed methods, these methods have proven to be challenging, lengthy, and rather inefficient. We have successfully used transposon insertion mutagenesis and plasmid-based complementation strategies to study gene function in *C. burnetii*; however, we have been unsuccessful making targeted mutations using described approaches, which led us to search for alternative methods to disrupt the function of specific genes in *C. burnetii*.

Genetic tools based on the clustered regularly interspaced short palindromic repeats (CRISPR) CRISPR-associated (Cas) (CRISPR-Cas) technology have substantially transformed genetic manipulation capabilities in a wide range of applications in biomedical research (45, 46). CRISPR interference (CRISPRi), which exploits the catalytically inactive Cas9 protein (dCas9) from *Streptococcus pyogenes* bound to a single guide RNA (sgRNA), has been developed as a genetic tool in both eukaryotic and prokaryotic cells to repress target gene expression via steric hindrance of RNA polymerase, thus preventing transcription initiation and/or elongation (47–49).

More recently, CRISPR-Cas9-mediated base editing emerged as a next-generation CRISPR tool that was engineered to efficiently and precisely edit DNA or RNA sequences within living cells (50–55). Two classes of DNA base editors have been described: adenine base editors (ABEs) that convert an A:T base pair to a G:C base pair, and cytosine base editors (CBEs) that convert a C:G base pair into a T:A base pair. Genome editing does not involve double-stranded DNA breaks and does not require a DNA repair template. Typically, DNA base editors utilize a fusion protein consisting of a catalytically impaired Cas nuclease, such as *S. pyogenes* Cas9 nickase (Cas9n), and a nucleobase deaminase enzyme that operates on single-stranded DNA. As with other CRISPR applications, sequence specificity is mediated by base pair complementarity between the Cas9n-bound sgRNA and the target DNA. Although base editing has been pioneered in eukaryotes, CBEs have been harnessed to generate targeted, unmarked loss-of-function mutants through the introduction of premature stop codons into open reading frames (ORFs) in various cell and animal models, including in human cells and in an increasing number of bacterial species (56–69). Premature termination of translation at introduced nonsense codons leads to the synthesis of truncated, likely non-functional proteins.

Here, we set out to expand the genetic toolbox for *C. burnetii* by describing two CRISPR-Cas9 technology-based approaches that enable the interrogation of *C. burnetii* gene function: CRISPRi-mediated gene silencing and CRISPR-Cas9-mediated cytosine base editing.

## RESULTS

### A CRISPRi approach to reduce gene expression in *C. burnetii*

**Fig 1:**
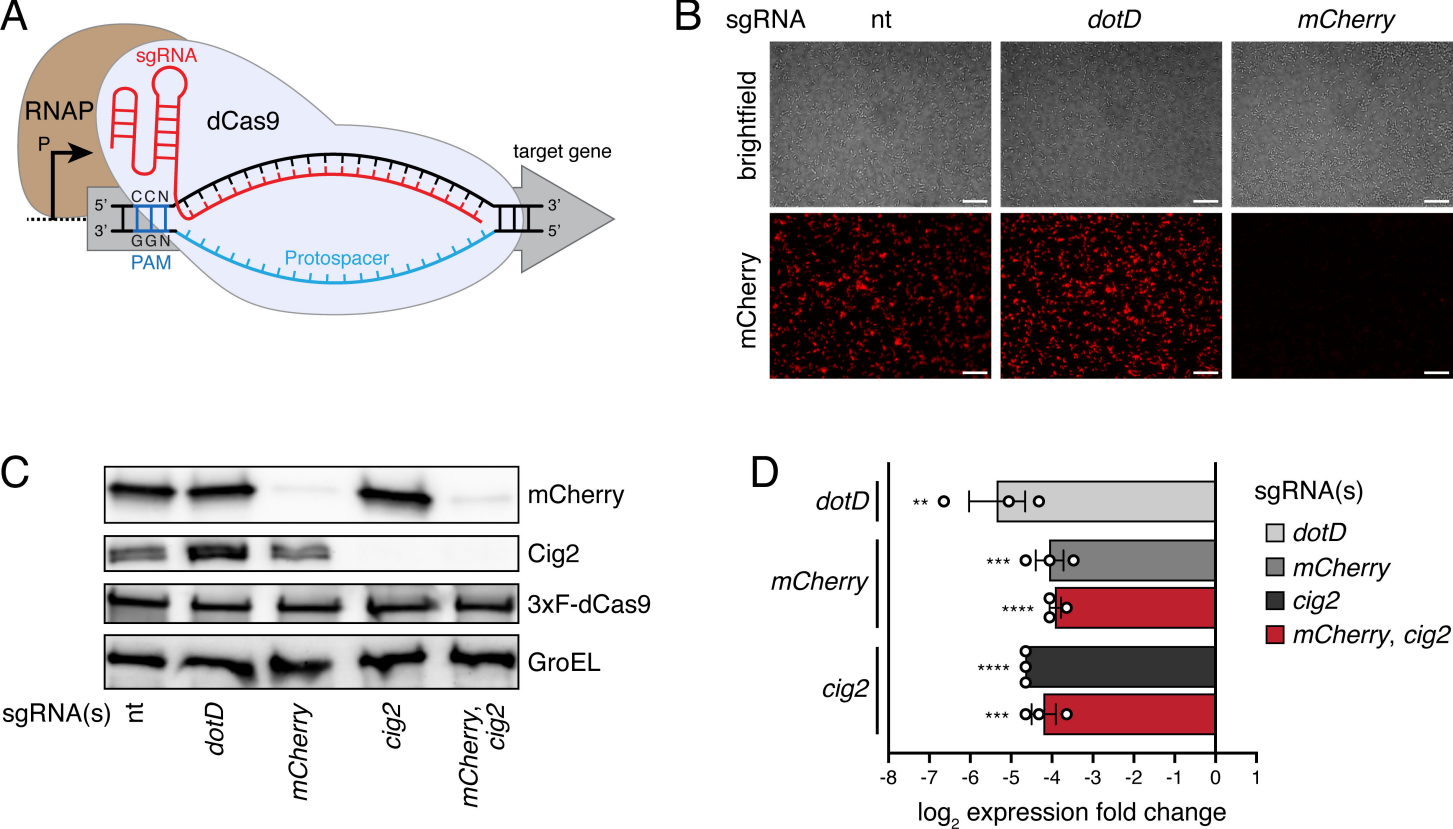
A plasmid-based CRISPRi system silences gene expression in *C. burnetii*, resulting in expected single gene and dual gene knockdown phenotypes in axenic medium. (**A**) Cartoon representation of CRISPRi-mediated interference of gene expression. The nuclease-deficient dCas9 (light blue) bound to a single guide RNA (sgRNA; red) targeting the non-template strand of a target gene (promoter region or early protein coding sequence) sterically precludes RNA polymerase (RNAP; brown) by acting as a roadblock for transcription initiation and/or elongation. The protospacer and NGG protospacer adjacent motif (PAM) are indicated. (**B**) Representative brightfield and mCherry fluorescence micrographs of the *mCherry*-expressing intergenic transposon mutant strain (ig::Tn) harboring individual pCB-CRISPRi plasmid derivatives expressing sgRNAs targeting indicated genes. Scale bars, 10 µm. nt, non-targeting. (**C**) Representative immunoblot analysis of protein levels of mCherry, Cig2, 3xF-dCas9, and GroEL (loading control) in ig::Tn strain harboring individual pCB-CRISPRi plasmid derivatives expressing sgRNAs targeting indicated genes (single gene or dual gene knockdown). nt, non-targeting. (**D**) mRNA expression levels (log_2_ expression fold change) in axenic medium of genes indicated on the left in ig::Tn strain harboring individual pCB-CRISPRi plasmid derivatives expressing sgRNAs targeting genes indicated by different colors in legend (single gene or dual gene knockdown). Means with SEs from three independent experiments are shown. A strain harboring a pCB-CRISPRi plasmid encoding either a non-targeting sgRNA or a sgRNA targeting a neutral gene was used as control sample, and the 16S rRNA gene served as endogenous reference gene for qRT-PCR and ∆∆Ct analysis. **P < 0.01; ***P < 0.001; ****P < 0.0001 by individual unpaired, two-tailed *t* tests (in comparison to corresponding control sample each).

We first asked whether a plasmid-based CRISPRi system could be used to modulate gene expression in *C. burnetii*. Interference with transcription initiation and/or elongation of target genes by dCas9/sgRNA complexes leads to gene-specific repression of gene expression (Fig. 1A). The plasmid-based CRISPRi system that was built for *C. burnetii* constitutively expressed both the N-terminally 3xFLAG-tagged *dCas9* from the *cbu1169* promoter and the gene-targeting sgRNA(s) from the synthetic bacterial promoter J23119(SpeI). For plasmid construction, a DNA template encoding the sgRNA was first introduced into helper plasmid pHelper-CRISPRi-sgRNA, and then the sgRNA-encoding region was moved to the *C. burnetii* CRISPRi plasmid pCB-CRISPRi (Fig. S1). The ability of this CRISPRi system to repress the expression of one gene individually or two genes simultaneously was examined using *C. burnetii* grown in axenic medium. Plasmid derivatives of pCB-CRISPRi encoding different sgRNAs were transformed into a strain of *C. burnetii* Nine Mile phase II (NMII) having a transposon insertion in a neutral intergenic region of the chromosome (ig::Tn). The transposon in this strain encodes *mCherry*, and intracellular replication of this strain is comparable to that of the wild-type *C. burnetii* NMII strain (14, 70). A strain containing a pCB-CRISPRi plasmid derivative expressing a sgRNA targeting the *mCherry* gene had both reduced mCherry fluorescence intensity and reduced mCherry protein level compared to isogenic strains expressing either a non-targeting sgRNA or a sgRNA targeting *dotD*, which encodes for an essential component of the Dot/Icm secretion system (71, 72) (Fig. 1B and C). A strain expressing a sgRNA targeting the gene encoding the Dot/Icm effector protein Cig2 displayed a specific absence of Cig2 protein (Fig. 1C). Furthermore, the co-expression of two sgRNAs in the same strain, one targeting *mCherry* and the other one *cig2*, resulted in severely reduced protein levels of both mCherry and Cig2, demonstrating that dual knockdowns can be achieved with this CRISPRi system (Fig. 1C). To examine the knockdown efficiencies of selected genes, quantitative reverse transcription polymerase chain reactions (qRT-PCRs) were performed using RNA isolated from axenically grown cells. The expression of the *dotD*, *mCherry*, and *cig2* genes was reduced by between 15- and 33-fold in the corresponding knockdown strains, including the *mCherry cig2* dual knockdown strain (Fig. 1D). Thus, this CRISPRi system successfully reduced expression of these *C. burnetii* genes, leading to expected phenotypes in axenic medium.

**Fig 2:**
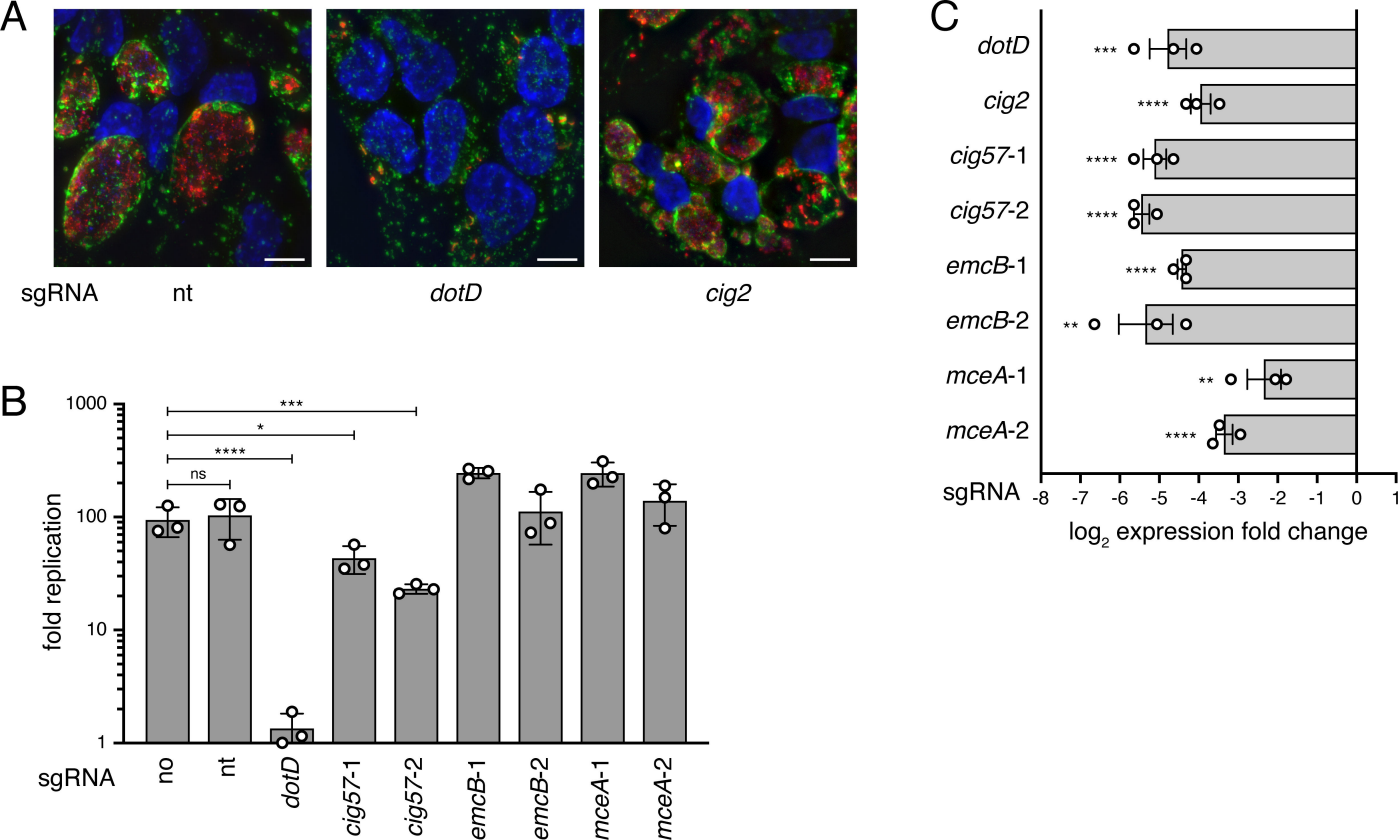
CRISPRi-mediated silencing of genes encoding for Dot/Icm secretion system components or effectors alters CCV biogenesis. (**A**) Representative immunofluorescence micrographs of HeLa cells infected with NMII wild-type harboring individual pCB-CRISPRi plasmid derivatives expressing sgRNAs targeting indicated genes for 5 days at an MOI of 300. Cells were fixed and stained with anti-LAMP-1 antibody (green), anti-*C*. *burnetii* antibody (red), and DAPI (blue). Scale bars, 10 µm. nt, non-targeting. (**B**) Intracellular replication of NMII wild-type harboring individual pCB-CRISPRi plasmid derivatives expressing sgRNAs targeting indicated genes is shown as fold replication (change in genome equivalents (GE) on day 7 post-infection relative to GE on the day of infection). THP-1 cells were infected at an MOI of 0.1. A representative data set is shown. Error bars are SDs. −1 and −2 indicate two separate sgRNAs targeting the same gene. no, pCB-CRISPRi plasmid without sgRNA construct; nt, non-targeting. *P < 0.05; ***P < 0.001; ****P < 0.0001; ns, not significant by one-way ANOVA with Bonferroni’s *post hoc* test. (**C**) mRNA expression levels (log_2_ expression fold change) in axenic medium of genes indicated on the left in NMII wild-type harboring individual pCB-CRISPRi plasmid derivatives expressing sgRNAs targeting genes indicated on the left. Means with SEs from three independent experiments are shown. Strains harboring either a pCB-CRISPRi plasmid without a sgRNA construct or a pCB-CRISPRi plasmid encoding a non-targeting sgRNA were used as control samples, and the 16S rRNA gene served as endogenous reference gene for qRT-PCR and ∆∆Ct analysis. −1 and −2 indicate two separate sgRNAs targeting the same gene. **P < 0.01; ***P < 0.001; ****P < 0.0001 by individual unpaired, two-tailed *t* tests (in comparison to corresponding control sample each).

To test whether this constitutive plasmid-based CRISPRi system can reproduce specific intracellular infection phenotypes that were reported previously for transposon insertion mutants, a selection of sgRNA-encoding pCRISPRi plasmid derivatives were transformed into wild-type *C. burnetii* NMII. As expected, compared to a strain expressing a non-targeting sgRNA, a strain expressing a sgRNA targeting *dotD* was incapable of creating large CCVs at 5 days post-infection in HeLa cells (Fig. 2A) and had a severe replication defect in THP-1 cells (Fig. 2B). Silencing the expression of *cig2* resulted in the appearance of multiple CCVs within infected cells, strongly resembling the so-called multivacuole phenotype that is attributed to strains deficient in Cig2 effector function (14, 15, 36) (Fig. 2A). Three additional genes, whose loss-of-function phenotypes had been described, were targeted individually by two separate sgRNAs each, and the intracellular replication of these strains was quantified in THP-1 cells. Compared to strains expressing either no or a non-targeting sgRNA, both strains expressing a sgRNA targeting the Dot/Icm effector protein-encoding gene *cig57* displayed reduced intracellular replication, whereas individually knocking down the expression of the effector-encoding genes *emcB* or *mceA* had no consistent effect on intracellular replication (Fig. 2B). The intracellular replication phenotypes displayed upon CRISPRi silencing of *cig57*, *emcB*, or *mceA* were similar to those reported in previous studies for transposon insertion mutants (14, 37, 73–75). The knockdown efficiencies of all genes targeted by CRISPRi in wild-type *C. burnetii* NMII were assessed by qRT-PCRs using RNA isolated from axenically grown strains. Individual sgRNAs led to a reduction in expression of *dotD*, *cig2*, *cig57*, and *emcB* by between 15- and 43-fold in corresponding knockdown strains, whereas the mRNA expression level of *mceA* was only reduced by approximately 5- to 10-fold (Fig. 2C).

Taken together, these results suggest that a plasmid-based CRISPRi system is a potential tool to study loss-of-function phenotypes of single or multiple genes of interest in *C. burnetii*. However, there was variability how efficiently CRISPRi was in silencing gene expression that could potentially prevent the uncovering of phenotypes associated with gene loss-of-function.

### Potential for targeted gene inactivation in *C. burnetii* using a CRISPR-Cas9-mediated cytosine base editing approach

**Fig 3:**
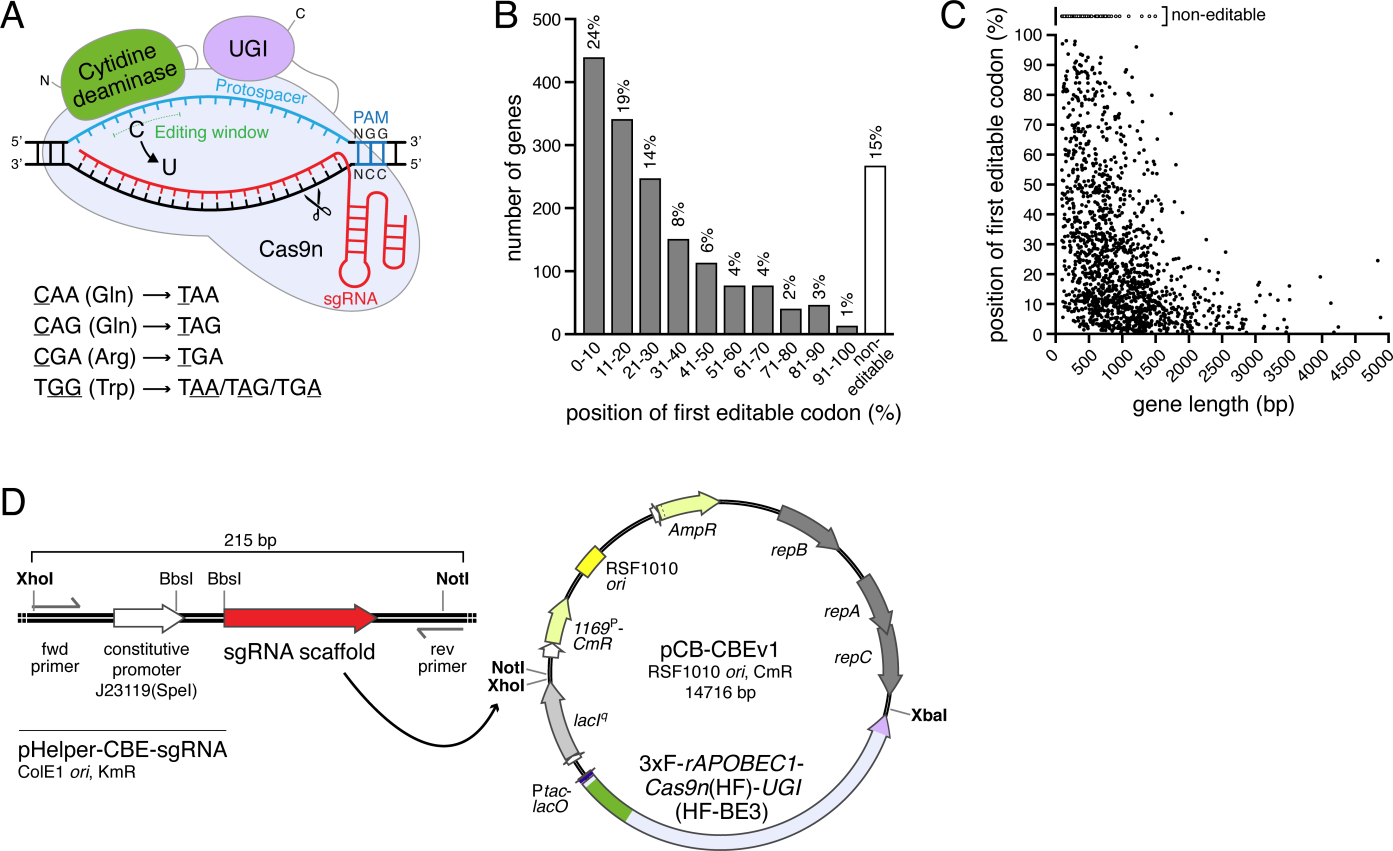
Using CRISPR-Cas9-mediated cytosine base editing to generate *C. burnetii* loss-of-function mutants by introducing premature stop codons into ORFs. (**A**) Cartoon representation of CRISPR-Cas9-mediated cytosine base editing. The CBE fusion protein consists of an N-terminal cytidine deaminase (APOBEC1; green), a central Cas9 nickase (Cas9n; light blue), and a C-terminal uracil DNA glycosylase inhibitor domain (UGI; light violet). The 5’ 20 bp sequence of a single guide RNA (sgRNA; red) bound to Cas9n directs the CBE to a complementary target sequence in the genome that is followed by an NGG protospacer adjacent motif (PAM). The cytidine deaminase gains access to the exposed DNA single strand and converts cytosines (C) that are positioned within the editing window to uracils (U). The editing window for the CBE HF-BE3 (76) used here typically is a five-nucleotide window that spans positions 4–8 of the protospacer (highest editing efficiency; counting the PAM as positions 21–23) (50). The UGI domain prevents repair of the deaminated cytidine by the endogenous DNA repair machinery. Aided by the activity of Cas9n that cleaves the non-edited strand, the U:G heteroduplex is permanently converted into a T:A base pair during subsequent DNA replication. The editing window, protospacer, and NGG protospacer adjacent motif (PAM) are indicated. The four different codons that can be converted into premature stop codons based on C to T (= G to A) substitutions mediated by CBEs are listed. The first three codons involve editing of the sense/coding strand; the last codon requires editing of the antisense/non-coding strand. (**B and C**) *In silico* genome analysis of ORFs annotated in *C. burnetii* Nine Mile RSA493 phase I (77) reveals the scope of CBE-mediated introduction of premature stop codons across the genome; 1,833 annotated protein-coding genes were analyzed, including essential genes, but excluding pseudogenes. (**B**) Histogram showing the total number of genes grouped by the relative position within each gene of their first option for premature stop codon introduction. Percentages shown above bars indicate fraction of total number of genes present in each group; 85% of *C. burnetii* NMI genes contain at least one option to introduce a premature stop codon. (**C**) Graph showing the relative position of the first option for premature stop codon introduction within each gene, as a function of gene length; 269 non-editable genes are shown as a function of gene length on top. (**D**) Plasmid maps of pHelper-CBE-sgRNA (left) and pCB-CBEv1 (right) used for CRISPR-Cas9-mediated cytosine base editing in *C. burnetii*. pHelper-CBE-sgRNA is a helper plasmid for CBE sgRNA cloning that harbors a sgRNA construct (targeting sequence followed by sgRNA scaffold with Cas9 handle; red) downstream of the synthetic, constitutive bacterial promoter J23119(SpeI) (Registry of Standard Biological Parts). Two BbsI sites allow the insertion of the desired 20 nt targeting sequence as a phosphorylated and annealed oligo duplex. Unique XhoI and NotI sites flank the sgRNA construct for restriction/ligation-mediated subcloning of the sgRNA-encoding region to pCB-CBEv1 (alternatively, the sgRNA-encoding region is moved to pCB-CBEv1 via PCR amplification and e.g. SLIC cloning). pCB-CBEv1 is the *C. burnetii* CBE plasmid (version 1) without sgRNA construct that encodes the CBE protein 3xF-rAPOBEC1-Cas9n(HF)-UGI (HF-BE3) downstream of the IPTG-inducible P*tac* promoter (however, basal expression is sufficient for base editing in *C. burnetii* using this plasmid). Plasmid contains unique XhoI, NotI, and XbaI sites for the introduction of sgRNA-encoding region(s) from pHelper-CBE-sgRNA helper plasmid derivatives (e.g. via restriction/ligation or SLIC cloning). Within the CBE-encoding gene, the cytidine deaminase-encoding sequence is highlighted in green, and the Cas9n- and UGI-encoding sequences are shown in light blue and light violet, respectively.

Considering abovementioned potential obstacles when performing CRISPRi experiments in *C. burnetii*, but building on the fact that dCas9/sgRNA complexes can be utilized for targeting specific genes in *C. burnetii*, we investigated whether a CRISPR-Cas9-mediated cytosine base editing approach could be leveraged to generate stable, unmarked loss-of-function mutants in *C. burnetii*. In this approach, a specific codon within a gene of interest is converted into a premature stop codon, which will result in the production of truncated, likely non-functional proteins. The type of CBE we chose for base editing in *C. burnetii* is based on the previously engineered and characterized high-fidelity CBE called HF-BE3 (76). This fusion protein consists of an N-terminal cytidine deaminase (APOBEC1), a central Cas9 nickase (Cas9n), and a C-terminal uracil DNA glycosylase inhibitor (UGI) domain (Fig. 3A). Bound to a sgRNA, the CBE is directed to a target sequence in the genome that is followed by an NGG protospacer adjacent motif (PAM). This results in cytosine to thymine transition mutations being generated within the editing window of the protospacer, leading to the conversion of a C:G base pair into a T:A base pair (Fig. 3A). Four codons can be changed into premature stop codons using cytosine base editing. These are the glutamine codons CAA and CAG, the arginine codon CGA, and the tryptophan codon TGG (Fig. 3A). To determine the overall utility of CBE-mediated gene inactivation in *C. burnetii*, an *in silico* genome analysis of all ORFs annotated in *C. burnetii* Nine Mile RSA493 phase I (NMI) (77) was performed using an online tool (78) (Fig. 3B and C). The data indicate that 85% of the 1,833 annotated protein-coding genes in *C. burnetii* could theoretically be inactivated by cytosine base editing as they contain at least one of the four codons within the required distance to an NGG PAM (Fig. 3B). According to this analysis, 43% of all ORFs (50% of all editable ORFs) are amenable to editing within the first 20% of the coding sequence, and 71% of all ORFs (83% of all editable ORFs) can be edited within the first 50% of the gene. As expected, the longer the gene, the higher the probability that a premature stop codon can be introduced by cytosine base editing within the first e.g. 20% or 50% of the ORF (Fig. 3C). There were 269 genes (15% of all ORFs) found to be non-editable, and 91% of these non-editable genes are shorter than 500 bp.

### Base-edited premature stop codon mutants display expected loss-of-function phenotypes

**Fig 4:**
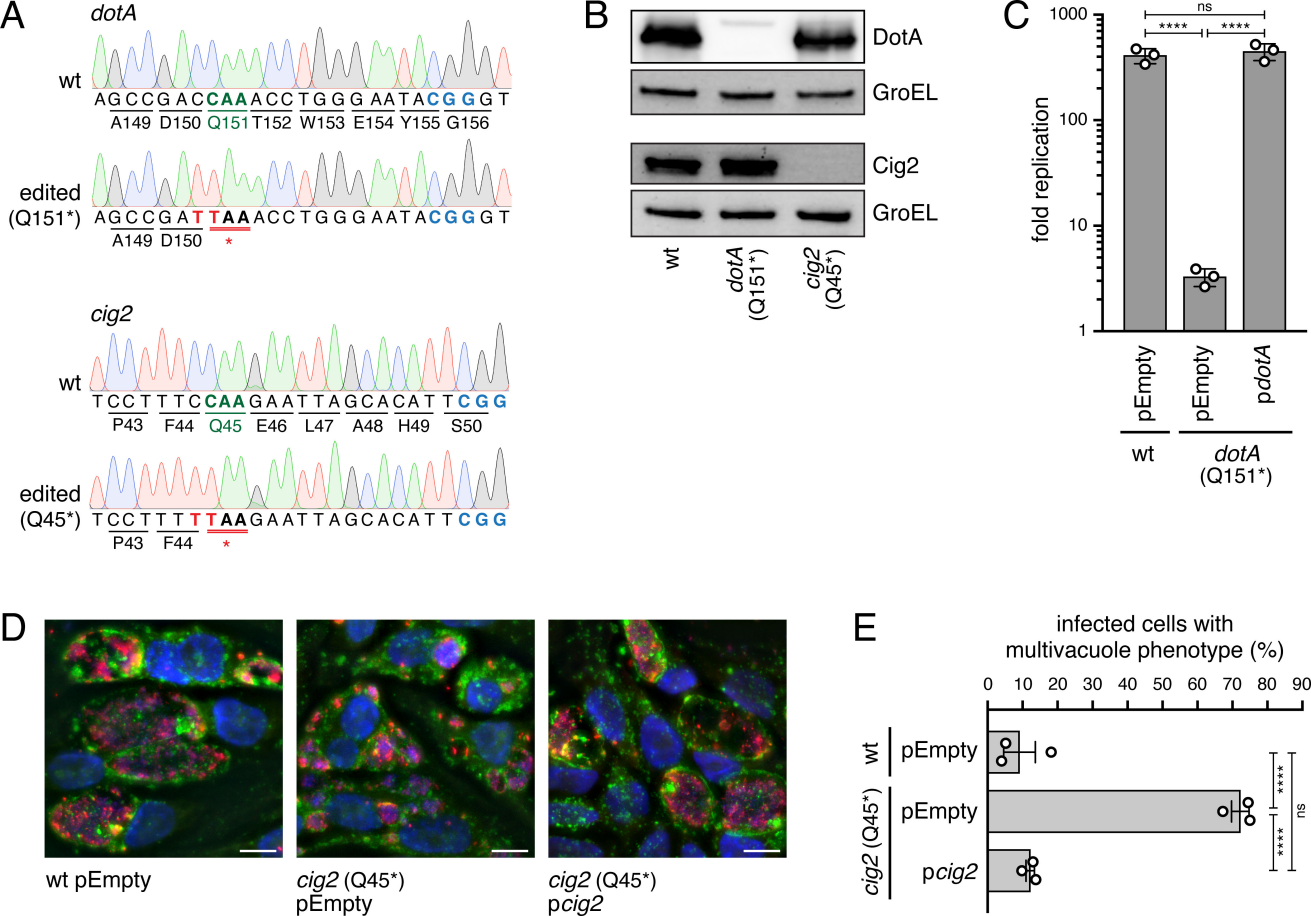
*C*. *burnetii* premature stop codon mutants constructed using CRISPR-Cas9-mediated cytosine base editing display distinct CCV biogenesis phenotypes. (**A**) Sanger sequencing chromatograms confirm successful base editing in the *C. burnetii* genome. Wild-type and edited loci of *dotA* (top) and *cig2* (bottom) are shown. Editable codons in wild-type sequences are shown in green, PAM sites are highlighted in blue, and thymines that were converted from cytosines are shown in red (C to T substitutions). Red horizontal double lines indicate introduced premature stop codons, resulting in Q151* in *dotA*, and Q45* in *cig2*. wt, wild-type. (**B**) Representative immunoblot analysis of protein levels of DotA, Cig2, and GroEL (loading control) in wild-type and indicated base-edited strains. wt, wild-type. (**C**) Intracellular replication of indicated strains is shown as fold replication (change in genome equivalents (GE) on day 6 post-infection relative to GE on the day of infection). THP-1 cells were infected at an MOI of 0.1. A representative data set is shown. Error bars are SDs. ****P < 0.0001; ns, not significant by one-way ANOVA with Tukey’s *post hoc* test. wt, wild-type. (**D**) Representative immunofluorescence micrographs of HeLa cells infected with indicated strains for 6 days at an MOI of 300. Cells were fixed and stained with anti-LAMP-1 antibody (green), anti-*C*. *burnetii* antibody (red), and DAPI (blue). Scale bars, 10 µm. wt, wild-type. (**E**) Quantification of the multivacuole phenotype (see [**D**]). Graph shows fraction of infected cells with multivacuole phenotype (more than two CCVs/cell) for indicated strains as means with SEs from three independent experiments. HeLa cells were infected for 5–6 days at a high MOI of 100–300. ****P < 0.0001; ns, not significant by one-way ANOVA with Tukey’s *post hoc* test. wt, wild-type.

To attempt cytosine base editing in *C. burnetii*, a plasmid was designed that encodes the N-terminally 3xFLAG-tagged CBE *HF-BE3* downstream of the IPTG-inducible P*tac* promoter. This *C. burnetii* CBE plasmid was named pCB-CBEv1 (Fig. 3D). Desired sgRNA-encoding regions were generated by first introducing a DNA template encoding the sgRNA into the helper plasmid pHelper-CBE-sgRNA downstream of the synthetic, constitutive bacterial promoter J23119(SpeI), before the sgRNA-encoding region was moved to pCB-CBEv1 to create a plasmid for cytosine base editing in *C. burnetii* (Fig. 3D). Constructs were created to generate premature stop codon mutants of *dotA* (Q151*; within the first 20% of the ORF), a gene encoding an essential component of the Dot/Icm secretion system (43), and *cig2* (Q45*; within the first 10% of the ORF), a gene coding for a Dot/Icm effector important for homotypic fusion of individual CCVs within infected cells (14, 15, 36). These individual CBE plasmids targeting *dotA* and *cig2* were electroporated into *C. burnetii*, and single colonies were isolated as described in Materials and Methods. For both genes, all tested colonies incorporated the targeted nonsense mutation. Sanger sequencing confirmed the successful editing of the *C. burnetii* genome (Fig. 4A). In both the base-edited *dotA* (Q151*) and *cig2* (Q45*) mutant strains, two consecutive cytosines within the editing window were converted to thymines, with the second one of the two being the one required for the introduction of the premature stop codon. Curing of the CBE plasmid from base-edited strains was achieved when a single colony of a strain containing a CBE plasmid was restreaked on non-selective ACCM-2 agarose. Over 50% of the resulting colonies were found to have lost the plasmid based on PCR analysis. An immunoblot analysis confirmed the Sanger sequencing results and showed that the *dotA* (Q151*) mutant and the *cig2* (Q45*) mutant did not produce the mutated protein of interest (Fig. 4B). Most importantly, both base-edited premature stop codon mutants displayed the expected phenotype during intracellular infection (Fig. 4C through E). The *dotA* (Q151*) mutant did not replicate in THP-1 cells, but this replication defect was reversed upon expression of wild-type *dotA* from a plasmid *in trans* (Fig. 4C), and the *cig2* (Q45*) mutant displayed a multivacuole phenotype, which is typical for infections with Cig2-deficient strains (14, 15, 36) (Fig. 4D and E). The multivacuole phenotype was complemented when wild-type *cig2* was introduced to the *cig2* (Q45*) mutant on a plasmid *in trans* (Fig. 4D and E). Taken together, these data demonstrate that CRISPR-Cas9-mediated cytosine base editing can be leveraged to generate *C. burnetii* loss-of-function mutants.

Because off-target mutations can occur in the genomes of bacteria mutated with the BE3 version of CBEs (50, 79, 80), whole genome sequencing was conducted on *dotA* (Q151*) and *cig2* (Q45*) mutant strains, and a single nucleotide polymorphism (SNP) analysis was performed to assess the number of off-target mutations. This analysis revealed the presence of a low level of spurious deamination in both strains (Table S1). Besides the desired base edit, 19 of 20 SNPs identified in the *dotA* (Q151*) mutant were C to T or G to A substitutions, which indicates that they are CBE-mediated off-target mutations. In the *cig2* (Q45*) mutant, 10 of 11 SNPs identified were CBE-mediated. Some of these mutations are nonsynonymous mutations, while others are synonymous, in between genes, or within pseudogenes. The analysis of whole genome sequencing data also identified five SNPs and one insertion and deletion (indel) that were present in all strains sequenced in this study, including the parental *C. burnetii* NMII wild-type strain (Table S2).

### A *cig57 cig2* double mutant displays an additive intracellular replication defect

**Fig 5:**
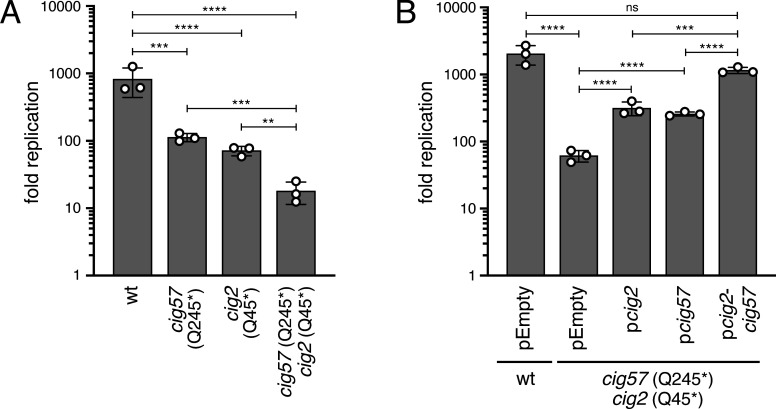
Combining premature stop codon mutations in *cig57* (Q245*) and in *cig2* (Q45*) leads to a severe additive intracellular replication defect that can be complemented. (**A and B**) Intracellular replication of indicated strains is shown as fold replication (change in genome equivalents (GE) on day 6 post-infection relative to GE on the day of infection). THP-1 cells were infected at an MOI of 0.1. Representative data sets are shown. Error bars are SDs. **P < 0.01; ***P < 0.001; ****P < 0.0001; ns, not significant by one-way ANOVA with Tukey’s *post hoc* test. wt, wild-type.

We next asked whether CRISPR-Cas9-mediated cytosine base editing could be used to create double mutants. The Dot/Icm effector-encoding genes *cig57* and *cig2* were selected for this analysis because they have unique and important roles during CCV biogenesis, but whether they function together or independently has not been determined (14, 15, 36, 37). Double mutants can either be constructed sequentially by transformation of an edited and plasmid-cured strain with a CBE plasmid targeting a second gene, or simultaneously by using a CBE plasmid that co-expresses sgRNAs targeting two different genes. Because of the potential advantage in saving time and effort, we asked whether a simultaneous base editing approach was feasible in *C. burnetii*. Thus, the approach was to generate a double mutant with premature stop codons in both *cig57* (Q245*; within the first 60% of the ORF) and *cig2* (Q45*; within the first 10% of the ORF) using a CBE plasmid expressing two sgRNAs. Screening of single colonies revealed that 67% of the tested colonies had incorporated both the *cig57* (Q245*) and *cig2* (Q45*) mutations, which indicates that the generation of double mutants in a single step is possible by cytosine base editing. Epistatic interactions between Cig57 and Cig2 were assessed by comparing the replication of isogenic single mutants generated by cytosine base editing to the double mutant in THP-1 cells. As expected, both the *cig57* (Q245*) and *cig2* (Q45*) single mutants displayed reduced replication compared to the parental *C. burnetii* NMII strain (Fig. 5A). Importantly, the *cig57* (Q245*) *cig2* (Q45*) double mutant showed an additive intracellular replication defect compared to either single mutant (Fig. 5A). Independently generated clones of base-edited strains displayed similar intracellular replication phenotypes, which suggests that potential off-target mutations were not complicating the analysis (Fig. S2). This was further supported by data showing that the intracellular replication defect of the *cig57* (Q245*) *cig2* (Q45*) double mutant was only partially reversed by expressing either wild-type *cig2* or *cig57* from a plasmid *in trans* and was fully complemented upon co-expression of both *cig2* and *cig57* from a plasmid *in trans* (Fig. 5B). These data demonstrate that a simultaneous cytosine base editing approach can be used for the introduction of premature stop codons to construct targeted gene inactivation double mutants in *C. burnetii*. Furthermore, given the additive phenotype observed for a strain with disrupted *cig57* and *cig2* ORFs, these results suggest that the two effectors Cig57 and Cig2 promote intracellular replication by separate, genetically independent pathways.

### A next-generation CBE causes fewer off-target edits

As described above, the specific loss-of-function phenotypes associated with strains that were edited using the CBE HF-BE3 could all be complemented by expressing corresponding wild-type alleles *in trans*. Nonetheless, the presence of off-target editing events in strains made with HF-BE3 (Table S1) prompted us to test the next-generation CBE BE4-PpAPOBEC1(H122A) (81) for base editing in *C. burnetii*. This previously characterized CBE functions with a similar on-target efficiency, but a minimized off-target activity compared to foundational CBEs like BE3/4 (81, 82). In comparison to the CBE HF-BE3 that consists of APOBEC1 from *Rattus norvegicus*, a high-fidelity Cas9 nickase, and one UGI domain (76), the improved CBE BE4-PpAPOBEC1(H122A) consists of high-fidelity APOBEC1(H122A) from *Pongo pygmaeus*, a Cas9 nickase, and two UGI domains (81). To test base editing in *C. burnetii* with the improved CBE, a plasmid was constructed that encodes the N-terminally 3xFLAG-tagged CBE *BE4-PpAPOBEC1*(H122A) downstream of the P*tac* promoter, so that the production of the CBE protein could be temporally regulated by IPTG. This next-generation *C. burnetii* CBE plasmid was named pCB-CBEv2 (Fig. S3). sgRNA-encoding regions were introduced into pCB-CBEv2 using the same helper plasmid approach as described for pCB-CBEv1 (Fig. S3). The genes *dotA* and *cig2* were targeted using the same sgRNAs to generate the stop codon mutations Q151* in *dotA*, and Q45* in *cig2*. Both premature stop codon mutants were successfully generated using the next-generation CBE plasmid. Depending on the duration of IPTG induction, approximately 33%–67% of the tested colonies obtained from transformations with the pCB-CBEv2 plasmids contained cells with the desired C to T substitution. In contrast to using pCB-CBEv1, IPTG induction was required for base editing when using pCB-CBEv2. Most importantly, whole genome sequencing of six base-edited strains from one experiment followed by a SNP analysis revealed the presence of very few CBE-mediated off-target mutations in these strains (Table S3). On average, strains edited with the improved CBE BE4-PpAPOBEC1(H122A) contained close to 10-fold fewer off-target edits compared to strains obtained with the CBE HF-BE3. Thus, the pCB-CBEv2 plasmid producing the BE4-PpAPOBEC1(H122A) protein has advantages over the pCB-CBEv1 plasmid producing the HF-BE3 protein for the generation of targeted loss-of-function mutations in *C. burnetii*.

## DISCUSSION

In this study, we demonstrate a CRISPRi approach that reduced the expression of most targeted genes over 15-fold in *C. burnetii*. All the *C. burnetii* CRISPRi knockdown strains displayed expected loss-of-function phenotypes in axenic medium and during intracellular infection. Concurrent to this study, two other research laboratories successfully applied the CRISPRi technology to repress gene expression in *C. burnetii*. Fu et al. employed a similar plasmid-based system that used constitutive expression of both the sgRNA and *dCas9* to silence the Dot/Icm effector-encoding gene *cirB* (83). Wachter et al. developed a chromosomally-encoded and IPTG-inducible CRISPRi system to silence individual QpH1 plasmid genes, genes encoding regulatory factors, and genes encoding Dot/Icm effectors (84–86). Cumulatively, this indicates that CRISPRi can be a useful tool to investigate gene function in *C. burnetii*. However, there are potential limitations to the technology as some genes may be more refractory to efficient CRISPRi silencing, and for some genes, low levels of gene expression may be sufficient for function. Therefore, we explored other approaches to generate stable loss-of-function mutants in *C. burnetii*.

Here, we demonstrate that CRISPR-Cas9-mediated cytosine base editing can be used to generate targeted *C. burnetii* loss-of-function mutants through the introduction of premature stop codons into genes of interest. In contrast to CRISPRi, which interferes with target gene transcription and requires the continuous expression of both *dCas9* and a sgRNA, the CRISPR-Cas9-mediated cytosine base editing approach generates stable mutants through inheritable gene inactivation. Both base editing proteins, the CBE HF-BE3 (76) and the next-generation CBE BE4-PpAPOBEC1(H122A) (81), were successful in generating targeted loss-of-function mutants in *C. burnetii*. Importantly, we found that *C. burnetii* double mutants could be generated in a single step by simultaneous base editing using a CBE plasmid that co-expresses sgRNAs targeting two different genes. The simultaneous modification of two or more loci is called multiplexing and saves time and effort compared to sequentially mutating genes. Based on these results, it is conceivable that a multiplexing approach could be used to target more than two genes at the same time in *C. burnetii*, as has been shown for other bacteria (64, 69). Elimination of the plasmid encoding the CBE protein was achieved in the mutated strains by restreaking a single colony on non-selective ACCM-2 agarose, which resulted in greater than 50% of colonies that were cured of the plasmid. Thus, the cytosine base editing protocol enables the rapid and reliable generation of targeted loss-of-function mutants in *C. burnetii*.

Importantly, base-edited premature stop codon mutants displayed expected loss-of-function phenotypes. Specifically, the *dotA* (Q151*) mutant did not replicate in THP-1 cells, and the *cig2* (Q45*) mutant displayed a multivacuole phenotype in HeLa cells. Similar to other approaches for generating loss-of-function mutants, off-target mutations can occur using a cytosine base editing approach due to spurious CBE-mediated deamination of cytosines. This did not pose a problem in *C. burnetii* as the phenotypes associated with individual base-edited strains could be complemented genetically by expressing wild-type copies of corresponding genes *in trans*. Additionally, the number of off-target mutations detected after mutagenesis was very low when using the next-generation CBE BE4-PpAPOBEC1(H122A) protein, which aligns with the high on-target/off-target editing ratio reported for BE4-PpAPOBEC1(H122A) (81).

The cytosine base editing approach applied here to generate *C. burnetii* loss-of-function mutants is based on the conversion of a specific codon (CAA, CAG, CGA, or TGG) within a gene of interest into a premature stop codon. An *in silico* analysis revealed that 85% of the annotated protein-coding genes in *C. burnetii* NMI are amenable to CBE-mediated introduction of a premature stop codon, thus demonstrating the overall utility of this approach for gene inactivation in *C. burnetii*. More than 80% of the editable ORFs can be terminated prematurely within the first 50% of the gene, which would likely prevent a functional protein from being produced. An important limitation of a base editing approach that employs *S. pyogenes* Cas9 is the strict requirement for a canonical NGG PAM at the correct distance from the editing site. Cas9 variants and other Cas enzymes with alternative and more relaxed PAM requirements have been reported (55, 87). For example, the recently described nearly PAM-less engineered Cas9 variant SpRY exhibits robust activity at sites with NRN PAMs, and a lower but substantial activity at sites with NYN PAMs (88). SpRY was further improved to SpRYc, which can specifically edit DNA at sites with diverse NNN PAMs (89). Thus, CBEs based on PAM-flexible Cas9 variants could potentially be used to edit *C. burnetii* genes that are not amenable to editing using the BE3 and BE4 CBE proteins used in this study.

**Fig 6:**
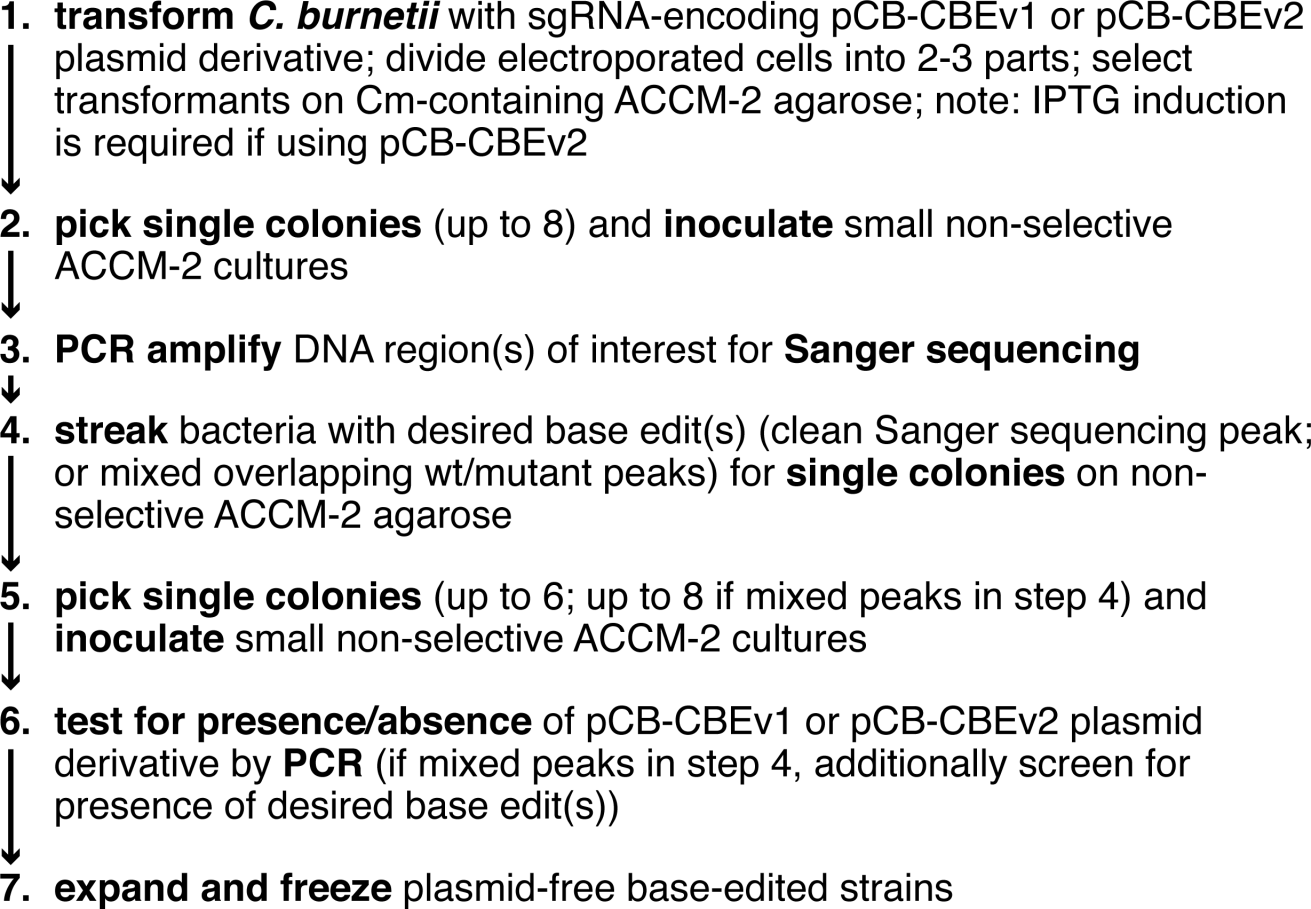
Overview of proposed workflow for CRISPR-Cas9-mediated cytosine base editing in *C. burnetii* using either pCB-CBEv1 (HF-BE3) or pCB-CBEv2 [BE4-PpAPOBEC1(H122A)]. In step 1, divide electroporated cells into two to three parts if independently generated base-edited strains are desired. In step 1, induction of *CBE* expression with 0.1 mM IPTG is required if using pCB-CBEv2, but not if using pCB-CBEv1 (e.g. for 48 h in liquid ACCM-2 before plating, or for an extended time in liquid ACCM-2 before plating and during growth on ACCM-2 agarose). See the Discussion and Materials and Methods for more details. Cm, chloramphenicol.

Based on our experience, we propose a simple and straightforward workflow for the construction of base-edited mutants in *C. burnetii* using either the CBE HF-BE3 or the CBE BE4-PpAPOBEC1(H122A) (Fig. 6). We suggest using *E. coli* XL1-Blue for the introduction of sgRNA-encoding region(s) into pCB-CBEv1 or pCB-CBEv2, and the maintenance of CBE plasmids. This *lacI^q^*-expressing strain ensures low basal production of the CBE proteins in *E. coli*. In step 1 of the proposed workflow, if the construction of independent clones of base-edited strains is desired, bacteria can be divided into different cultures immediately after the electroporation-based transformation with the CBE plasmid. IPTG induction is not necessary when using pCB-CBEv1; however, IPTG induction is required for base editing with pCB-CBEv2. The duration of IPTG induction likely affects the on-target/off-target editing ratio, so the duration of induction should be determined empirically for each target gene of interest. In step 2, selecting and analyzing eight individual single colonies from the transformation plate is in most cases sufficient to isolate a desired base-edited mutant. For each independent transformation, the percentage of colonies that harbor the mutation(s) of interest depends on the time at which base editing occurs. For example, if base editing occurs efficiently and early during *C. burnetii* growth in the liquid culture before plating, a high percentage of individual colonies on the transformation plate will likely contain the desired mutation(s), but these colonies may also more likely be siblings that all have the same off-target mutations. It is also possible that base editing occurs during the formation of a colony on the transformation plate, which results in both the wild-type and mutant nucleotide being detected at the desired position in a Sanger sequencing chromatogram (step 3). In step 4, bacteria with desired mutation(s) are streaked for single colonies on an ACCM-2 agarose plate without antibiotic selection, which ensures the clonal isolation of the desired base-edited mutant detected in the Sanger sequencing chromatogram. These colonies are also tested by PCR for the loss of the CBE plasmid (steps 4–6). Loss of the CBE plasmid occurs very frequently as more than 50% of colonies are generally cured of the plasmid following one restreak on non-selective ACCM-2 agarose. In step 7, base-edited, plasmid-free strains are expanded and frozen. The overall editing efficiencies observed at specific loci in the *C. burnetii* genome are likely influenced by factors shown to be important for CRISPR-Cas9 technology-based approaches, such as the accessibility of target gene loci or the sequence composition of the sgRNAs used (90, 91).

We used cytosine base editing to generate a double mutant deficient in *cig57* and *cig2*, two genes encoding Dot/Icm effector proteins with important roles during CCV biogenesis and intracellular replication. This represents an important advance, as this is to the best of our knowledge, the first time a specific effector double mutant has been generated in *C. burnetii*. The availability of loss-of-function mutants deficient in two or more effector genes is instrumental to the investigation of epistatic relationships among effector proteins, which will enable genetic studies to determine whether effectors function together or independently from one another. Our data revealed an additive intracellular replication defect for a *cig57* (Q245*) *cig2* (Q45*) double mutant in THP-1 cells, when this double mutant was compared to either isogenic single mutant. Expressing either wild-type *cig2* or *cig57* individually in the *cig57* (Q245*) *cig2* (Q45*) double mutant resulted in partial rescue, whereas, co-expression of both *cig2* and *cig57* fully rescued the replication defect of the double mutant. These data are consistent with the effector proteins Cig57 and Cig2 functioning on two different host cellular pathways that contribute to intracellular replication, rather than by acting together to modulate one pathway. The observed intracellular replication defect of a *cig57* (Q245*) single mutant in macrophage-like cells agrees with previously reported findings in HeLa cells using a mutant with a transposon insertion in *cig57* (14, 37). In our experiments, the base-edited *cig2* (Q45*) single mutant also displayed reduced replication in THP-1 cells compared to the parental *C. burnetii* NMII strain, which is consistent with the replication defect in THP-1 cells observed for a ∆*cig2* deletion mutant and a strain in which *cig2* was silenced by CRISPRi (86, 92). Two independent studies have shown that *cig2* transposon insertion mutants do not show intracellular growth defects in HeLa cells and other epithelial cell lines, even though a robust multivacuole phenotype is observed in these cells (14, 36). It is thus likely that the contribution of Cig2 to optimal intracellular replication of *C. burnetii* varies between host cells. There is substantial knowledge on the molecular functions of Cig57 and Cig2. Cig57 interacts with FCHO2, an early-arriving component of clathrin-coated vesicles, thus promoting the recruitment of clathrin to the CCV (37). Clathrin-coated vesicles may act as a source of membrane or nutrients once these vesicles fuse with the CCV. Consistent with this hypothesis, *C. burnetii* replication is strongly impaired in clathrin-depleted cells (37, 93). Cig2 not only mediates homotypic fusion between individual CCVs in a cell but also plays a critical role for the fusion of the CCV with autophagosomes (14, 15, 36). Cig2 binds to the phosphatidylinositol 3-phosphate (PI3P)-rich CCV membrane, where it was suggested to modulate phosphoinositide metabolism possibly by interfering with the activity of the phosphatidylinositol 5-kinase PIKfyve to enable an enrichment of PI3P on CCV membranes, which would then favor the recruitment of the autophagy machinery that mediates CCV homotypic fusion (36). CCVs formed by both the *cig2* and *cig57* mutant are impaired in fusing with autophagosomes as they do not accumulate the autophagy protein LC3 (14, 15, 36, 94). Also, CCVs of both mutants do not stain positive for clathrin (37, 94). Despite these apparent links between Cig57- and Cig2-mediated biology, our data revealed an additive intracellular replication defect of a *cig57 cig2* double mutant in THP-1 cells, suggesting that the two effectors regulate *C. burnetii* replication by modulating distinct pathways.

In conclusion, we have successfully adapted two CRISPR-Cas9-based approaches to study gene function in *C. burnetii*, CRISPRi to silence gene expression and CRISPR-Cas9-mediated cytosine base editing to construct targeted loss-of-function mutants. While CRISPRi has been applied in *C. burnetii* before, this study for the first time describes the implementation of CRISPR-Cas9-mediated cytosine base editing to make mutants in *C. burnetii*. Applying this cutting-edge technology for genome engineering with its simplicity and high efficiency will advance future studies on the obligate intracellular pathogen *C. burnetii* and its interaction with host cells.

## MATERIALS AND METHODS

### Bacterial strains, cell lines, and growth conditions

Bacterial strains, plasmids, and primers used in this study are listed in Table S4. *C. burnetii* Nine Mile RSA439 (phase II, clone 4) (NMII) (95), and derivatives were cultured axenically in liquid acidified citrate cysteine medium 2 (ACCM-2; Sunrise Science Products) for 5–8 days or on ACCM-2 agarose plates (ACCM-2 with 1% UltraPure Agarose [Invitrogen], supplemented with 0.5 mM L-tryptophan) for >8 days at 37°C, 5% CO_2_, and 2.5% O_2_ as previously described (38, 39, 96). When appropriate, kanamycin (375 µg/mL) and/or chloramphenicol (3 µg/mL) were added to ACCM-2. *C. burnetii* genome equivalents (GE) were enumerated by quantitative PCR (qPCR) using *dotA*-specific primers as previously described (14). *E. coli* strains were routinely grown at 37°C in Luria Bertani (LB) broth or on LB agar plates supplemented with appropriate antibiotics (ampicillin [100 µg/mL]; kanamycin [50 µg/mL]; chloramphenicol [25 µg/mL]).

HeLa cells (ATCC CCL-2) were routinely grown in Dulbecco’s Modified Eagle’s Medium (DMEM) supplemented with 10% heat-inactivated fetal bovine serum (FBS) at 37°C in 5% CO_2_. THP-1 monocytes were maintained in Roswell Park Memorial Institute (RPMI) Medium 1640 (ATCC modification; Gibco, A1049101) supplemented with 50 µM β-mercaptoethanol and 10% heat-inactivated FBS at 37°C in 5% CO_2_.

### Plasmid constructions and *C. burnetii* transformations

Plasmids were constructed by standard restriction/ligation cloning, sequence- and ligation-independent cloning (SLIC), or Gibson assembly. See Table S4 for plasmids and primers used in this study, and details on plasmid constructions. The following plasmids were deposited to Addgene: pHelper-CRISPRi-sgRNA (Addgene plasmid #221135), pCB-CRISPRi (Addgene plasmid #221136), pHelper-CBE-sgRNA (Addgene plasmid #221137), pCB-CBEv1 (Addgene plasmid #221138), and pCB-CBEv2 (Addgene plasmid #221139). Plasmid constructions involved the use of several published plasmids: psgRNA-base (97), pVdCas9hum_RBSmut1 (97), pET42b-HF-BE3 (76), pCMV-BE4-PpAPOBEC1 H122A (81), pJB-KAN-3xFLAG (41), pJB-CAT-3xFLAG (41), pJB-KAN-TetRA-3xFLAG (98), pMiniTn7T-KAN-L3S2P21 (15, 99), pJC-CAT (43), pJB1806 (100), pMTX808 (101), and pSAST207 (70).

Plasmids were transformed into *C. burnetii* by electroporation. For each transformation, recipient strain was grown in 10 mL ACCM-2 for 5–6 days. On ice, cells were washed twice with ice-cold sterile 10% glycerol (5 mL, 1 mL; 3,000 × *g*, 15 min, 4°C), and then resuspended in 50 µL 10% glycerol. Competent cells were mixed with 7–10 μg plasmid DNA in a 0.1 cm electroporation cuvette (Bio-Rad). Following electroporation with an Electro Cell Manipulator ECM630 (BTX Harvard Apparatus) (settings: 1.8 kV, 500 Ω, 25 µF), cells were resuspended in 950 µL RPMI Medium 1640, and 200 µL of this mix were moved to 3 mL ACCM-2 in a 6-well plate. Cells were grown for 24 h before selective antibiotics were added (kanamycin [375 µg/mL] or chloramphenicol [3 µg/mL]). Cells were grown for 4 days before they were collected and concentrated by centrifugation (3,000 × *g*, 15 min). Cells were applied onto a small area of an ACCM-2 agarose plate (containing appropriate antibiotics) and streaked for single colonies. Single colonies were picked and expanded in liquid ACCM-2. Immunoblots were generally used to confirm the production of 3xFLAG-tagged proteins in *C. burnetii* transformants.

### CRISPR-Cas9-mediated cytosine base editing

To construct base-edited premature stop codon mutants, sgRNA-encoding pCB-CBEv1 and pCB-CBEv2 plasmid derivatives were transformed into wild-type *C. burnetii* NMII by electroporation as described above. See Table S4 for details on plasmids used. Strain constructions using pCB-CBEv1 were done without IPTG induction, as basal *CBE* expression was sufficient for base editing.

The *dotA* (Q151*) (SS448) and *cig2* (Q45*) (SS446) mutants were constructed using plasmids pSAST359 and pSAST373, respectively. To estimate the editing efficiency, approximately six colonies each were picked from the chloramphenicol-containing ACCM-2 agarose transformation plate to inoculate individual 1 mL ACCM-2 cultures (supplemented with chloramphenicol). Cultures served as PCR templates to amplify and Sanger sequence *dotA* or *cig2*. The editing efficiency was high for both genes, as all colonies tested had clean Sanger sequencing peaks showing the desired C to T substitution. One to two colonies each were picked from the transformation plate and streaked for single colonies on non-selective ACCM-2 agarose. Ten days later, 12 colonies of each restreak were picked to inoculate non-selective 1 mL ACCM-2 cultures. Cultures served as PCR templates to test for the presence/absence of CBE plasmids. More than 50% of cultures were plasmid-free. Loci of interest were re-sequenced to confirm base editing.

The two independent *cig57* (Q245*) mutants (SS454 and SS455) were constructed using plasmid pSAST369. Plasmid pSAST370 was used to construct the second independently generated *cig2* (Q45*) mutant (SS457). To get independent clones of base-edited strains, electroporated cells were divided into two parts by moving 2 × 200 µL from the cuvette to 3 mL ACCM-2. Two to three colonies each were picked from the chloramphenicol-containing ACCM-2 agarose transformation plate and streaked for single colonies on non-selective ACCM-2 agarose. Five days later, four colonies of each restreak were picked to inoculate non-selective 1 mL ACCM-2 cultures. Cultures served as PCR templates to test for the presence/absence of CBE plasmids. Approximately 50% of cultures were plasmid-free. For each divided transformation, the gene of interest of one plasmid-free clone was amplified and sequenced. For *cig57*, both independent clones displayed the desired base edit, while for *cig2*, one of the two strains was successfully edited.

The two independent *cig57* (Q245*) *cig2* (Q45*) double mutants (SS488 and SS490) were constructed by simultaneous base editing using plasmid pSAST394. To get independently generated clones, electroporated cells were divided into three parts as described above. Two colonies each were picked from the transformation plate and streaked for single colonies on non-selective ACCM-2 agarose. Ten days later, two colonies of each restreak were picked to inoculate non-selective 1 mL ACCM-2 cultures. Cultures served as PCR templates to test for the presence/absence of CBE plasmids. More than 50% of cultures were plasmid-free. Both *cig57* and *cig2* genes were amplified and sequenced from a total of six plasmid-free clones (two each from each divided transformation). Four of the six clones (originating from two of the three parts of the divided transformation) displayed the desired base edits in both genes, suggesting a high editing efficiency.

To construct the three *dotA* (Q151*) (SS504, SS499, SS500) and three *cig2* (Q45*) (SS497, SS495, SS496) mutants made with the improved CBE BE4-PpAPOBEC1(H122A) encoded on pCB-CBEv2, plasmids pSAST383 and pSAST382 were transformed into wild-type *C. burnetii* NMII as described above. Electroporated cells were divided into three parts by moving 3 × 200 µL from the cuvette to 3 mL ACCM-2. For the first part, the *CBE* expression was induced by the addition of 0.1 mM IPTG to the liquid culture 24 h after transformation (extended IPTG induction). The second part was only induced 48 h before plating (48 h IPTG induction), and the third part was left uninduced. Bacteria from both the uninduced and 48 h IPTG induction conditions were plated on chloramphenicol-containing ACCM-2 agarose plates, cells from the extended IPTG induction condition were plated on ACCM-2 agarose plates containing chloramphenicol and 0.1 mM IPTG. Three colonies each were picked from the transformation plate and streaked for single colonies on non-selective ACCM-2 agarose. Eight days later, four colonies of each restreak were picked to inoculate non-selective 1 mL ACCM-2 cultures. Cultures of a total of 36 clones originating from each initial transformation served as PCR templates to amplify and Sanger sequence *dotA* or *cig2*. No editing was observed without IPTG induction. For both genes in average, 33% of colonies from the 48 h IPTG induction condition displayed the desired base edit (originating from approximately 33% positive [or mixed] colonies on transformation plates), and 58% of colonies from the extended IPTG induction condition had the desired mutation (originating from approximately 67% positive [or mixed] colonies on transformation plates). Base-edited strains were tested by PCR for the presence/absence of CBE plasmids. More than 50% were plasmid-free.

### *C. burnetii* infections and GE-based intracellular replication assays

For *C. burnetii* intracellular replication assays in THP-1 cells, monocytes were plated into 24-well plates at a density of 5 × 10^5^ cells/well and differentiated into macrophage-like cells in the presence of 200 nM phorbol 12-myristate 13-acetate (PMA) for approximately 44 h before they were infected at a multiplicity of infection (MOI) of 0.1 with indicated strains. MOI calculations were done based on *C. burnetii* GE enumerated by qPCR using *dotA*-specific primers. Infections were synchronized by spinning the plates (600×*g*, 15 min, 30°C). At 1 h post-infection, cells were washed twice with PBS before the addition of fresh medium. Immediately afterwards (day 0) and at other indicated time points post-infection, THP-1 cells were lyzed with deionized H_2_O, and lysate was combined with well supernatant. *C. burnetii* was pelleted (20,000 × *g*, 10 min), and pellets were frozen at −20°C until further processing. Total genomic DNA was extracted from pellets using glass beads and a Bullet Blender as previously described (85). Briefly, 150 µL nuclease-free deionized H_2_O and one scoop (0.1 g) of 0.1 mm glass beads (Next Advance) were added to pellets before samples were bead-beaten using a Bullet Blender BBX24 (Next Advance) (settings: speed 8, 4 min). Samples were centrifuged (20,000×*g*, 1 min), boiled for 10 min, and centrifuged again (20,000 × *g*, 1 min). Supernatants (80 µL) were collected and GE were quantified by qPCR using *dotA*-specific primers. *Cig2*-specific primers were used in *dotA* complementation experiment.

### RNA isolation and qRT-PCRs

Strains harboring individual pCB-CRISPRi plasmid derivatives were grown in ACCM-2 for 7–8 days before GE were enumerated by qPCR using *dotA*-specific primers. Fresh 10 mL ACCM-2 cultures were inoculated at a concentration of 1.66 × 10^7^ GE/mL and grown for 3 days before bacteria were pelleted (3,000 × *g*, 15 min). Cells were resuspended in 500 µL RNAprotect Bacteria Reagent (Qiagen) and incubated for 5 min at room temperature. Bacteria were pelleted again (20,000 × *g*, 10 min), and pellets were snap-frozen in liquid nitrogen for storage at −80°C until further processing. RNA isolation and purification were performed using the RNeasy Mini Kit (Qiagen). Before adding 350 µL Buffer RLT supplemented with 143 mM β-mercaptoethanol, bacteria were resuspended in 100 µL RNase-free TE buffer (Invitrogen) containing 15 mg/mL lysozyme and incubated for 5 min at room temperature. During the RNA cleanup, an on-column DNase digestion step was performed using double-strength RNase-free DNase (Qiagen). RNA was eluted with 80–120 μL RNase-free H_2_O, and 250 ng RNA were reverse transcribed with the iScript Advanced cDNA Synthesis Kit (Bio-Rad). The abundance of cDNA for genes of interest was determined by qPCR using the iQ SYBR Green Supermix (Bio-Rad) and a Bio-Rad CFX thermocycler. Primers used for qRT-PCRs are listed in Table S4. qRT-PCR data were analyzed by the ∆∆Ct method (102), using the 16S rRNA gene as the endogenous reference gene.

### Fluorescence microscopy

#### Immunofluorescence microscopy

HeLa cells were seeded into 24-well plates containing glass coverslips at a density of 4 × 10^4^ cells/well in DMEM 5% FBS at least 8 h before they were infected with indicated strains at indicated MOIs. Approximately 24 h post-infection, cells were washed once with PBS before the addition of fresh DMEM 5% FBS. At indicated time points post-infection, cells were washed once with PBS and fixed with 4% paraformaldehyde (PFA) for 15 min at room temperature. Cells were rinsed three times with PBS, permeabilized and blocked in PBS containing 0.05% saponin and 2% bovine serum albumin (BSA) for 1 h at room temperature. Coverslips were incubated with primary antibodies (mouse anti-LAMP-1 [1:500] [Developmental Studies Hybridoma Bank at the University of Iowa, H4A3] and rabbit anti-*C*. *burnetii* [1:5,000] [24]) diluted in permeabilization and blocking solution for 1 h, rinsed with PBS three times, and incubated with appropriate secondary antibodies (Alexa Fluor goat anti-mouse 488 [1:2,000] and Alexa Fluor goat anti-rabbit 568 [1:2,000] [Invitrogen]) diluted in permeabilization and blocking solution for 1 h at room temperature; 0.2 µg/mL DAPI (Sigma) was used to stain host and bacterial DNA. Coverslips were washed three times with PBS and mounted onto glass slides using ProLong Gold antifade reagent (Invitrogen). Fluorescence micrographs were acquired with a Nikon Eclipse TE2000-S inverted fluorescence microscope equipped with a CoolSNAP EZ camera from Photometrics and a Nikon Plan Apo100× objective lens (1.4 numerical aperture) under the control of SlideBook 6.0 software (Intelligent Imaging Innovations). For the quantification of the multivacuole phenotype, approximately 50 infected cells from random fields of view were scored for each strain in each experiment.

#### Live fluorescence microscopy

Indicated strains were grown in ACCM-2 for 7 days before they were concentrated by centrifugation (20,000 × *g*, 5 min); 2–3 μL of the concentrated bacterial suspension was applied onto a 1% low melting agarose (G-Biosciences) pad sandwiched between a glass slide and a coverslip (103). Live fluorescence and brightfield micrographs were acquired using abovementioned microscope.

### Immunoblotting

Indicated strains were grown in ACCM-2 for 7 days. Bacteria were pelleted (3,000 × *g*, 15 min), concentrated approximately 4-fold by resuspending them in 2 mL PBS, and OD_600_ measurements were used to adjust strains to the same concentration. Samples for anti-DotA immunoblot were not boiled before loading the gel to prevent DotA protein aggregation (104). Proteins on immunoblots were detected using primary mouse anti-FLAG (1:10,000) (Sigma, F3165), rabbit anti-mCherry (1:1,000) (BioVision, 5993), rabbit anti-GroEL (1:5,000) (CUSABIO, CSB-PA323108XA01DXP), rabbit anti-Cig2 (1:1,000) (Roy Lab, unpublished), and rabbit anti-DotA (1:1,000) (105) (kind gift from Edward I. Shaw [PCOM South Georgia]) antibodies and HRP-conjugated goat anti-mouse and goat anti-rabbit secondary antibodies (1:5,000) (Invitrogen).

### Whole genome sequencing and SNP analysis

Total genomic DNA from *C. burnetii* strains was extracted using the Bacteria genomicPrep Mini Spin Kit (Cytiva). A 15 min RNase A treatment was done during DNA purification. Paired-end Illumina sequencing was performed by the Yale Center for Genome Analysis (YCGA). Whole genome sequencing data were analyzed using the Galaxy online platform (106). Reads were mapped against the *C. burnetii* Nine Mile RSA439 (phase II, clone 4) reference genome (GenBank accession numbers: CP020616.1 [chromosome]; CP020617.1 [plasmid] [107]). The following Galaxy tools were run sequentially with indicated settings: BWA-MEM2 (paired-end reads; simple Illumina mode; sort by chromosomal coordinates), Samtools mpileup (basic options; default output options), and VarScan mpileup (run individually for single nucleotide variation analysis and for insertions and deletions analysis; default settings (minimum coverage: 8; minimum supporting reads: 2; minimum base quality: 15); minimum variant allele frequency (VAF) was set to 0.75). SNPs and indels identified in all strains sequenced in this study (including wild-type *C. burnetii* NMII [SS364]) were confirmed by Sanger sequencing.

### *In silico* genome analysis of editable codons

The CRISPR RGEN BE-Designer online tool (78) was used to test whether *C. burnetii* genes are amenable to CBE-mediated introduction of a premature stop codon and to determine the first option for the introduction of a premature stop codon along the length of a gene. The *in silico* tool was run gene by gene for all 1,833 annotated protein-coding ORFs (including essential genes, but excluding pseudogenes) of *C. burnetii* Nine Mile RSA493 phase I (GenBank accession numbers: AE016828.3 [chromosome]; AE016829.2 [plasmid] [77]) with the following settings: SpCas9 from *Streptococcus pyogenes*: 5′-NGG-3′ (CRISPR-Cas orthologue for base editing); 20 (crRNA length); BE (C to T) (base editing type); 13–17 (which is 4–8 if the PAM is counted as positions 21–23) (base editing window). Twenty nucleotides up- and downstream each ORF were included in input sequences.

### Statistical analysis

Statistical analyses were conducted using GraphPad Prism (GraphPad Software). *P* values were calculated by unpaired, two-tailed *t* tests, or by one-way ANOVA with Tukey’s or Bonferroni’s *post hoc* test as indicated in figure legends. *P* values < 0.05 were considered significant.

## SUPPLEMENTAL MATERIAL

Supplemental material msphere.00523-24-s0001.pdf Fig. S1–S3; Tables S1–S3.

Table S4 msphere.00523-24-s0002.xlsx Strains, plasmids, and primers.

ASM does not own the copyrights to Supplemental Material that may be linked to, or accessed through, an article. The authors have granted ASM a non-exclusive, world-wide license to publish the Supplemental Material files. Please contact the corresponding author directly for reuse.
