# Supplementary material for: CRISPR-Cas9-based approaches for genetic analysis and epistatic interaction studies in Coxiella burnetii
Source: mSphere. 2024 Nov 19;9(12):e00523-24. doi: 10.1128/msphere.00523-24 (PMC11656778; doi:10.1128/msphere.00523-24)
Supplement: Supplemental material — Fig. S1–S3; Tables S1–S3. [file msphere.00523-24-s0001.pdf]

Supplemental Material

CRISPR-Cas9-based approaches for genetic analysis and epistatic interaction studies in *Coxiella burnetii*

Samuel Steiner, Craig R. Roy

Figure S1

Figure S2

Figure S3

Table S1

Table S2

Table S3

Table S4 (separate file)

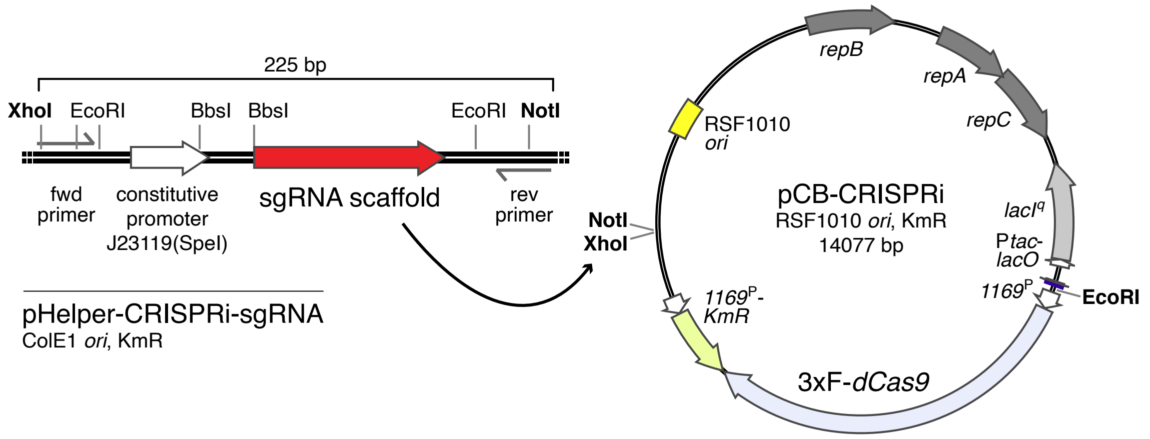

Figure S1 Additional data related to Figure 1. Plasmid maps of pHelper-CRISPRi-sgRNA (left) and pCB-CRISPRi (right) used for plasmid-based CRISPRi-mediated interference of gene expression in *C. burnetii*. pHelper-CRISPRi-sgRNA is a helper plasmid for CRISPRi sgRNA cloning that harbors a sgRNA construct (targeting sequence followed by sgRNA scaffold with dCas9 handle; red) downstream of the synthetic, constitutive bacterial promoter J23119(SpeI) (Registry of Standard Biological Parts). Two BbsI sites allow the insertion of the desired 20 nt targeting sequence as a phosphorylated and annealed oligo duplex. Unique XhoI and NotI sites (or EcoRI sites) flank the sgRNA construct for restriction/ligation-mediated subcloning of the sgRNA-encoding region to pCB-CRISPRi (alternatively, the sgRNA-encoding region is moved to pCB-CRISPRi via PCR amplification and e.g. SLIC cloning). pCB-CRISPRi is the *C. burnetii* CRISPRi plasmid without sgRNA construct that encodes 3xF-dCas9 downstream of the *cbu1169* promoter (*1169*^{P}). Plasmid contains unique XhoI, NotI, and EcoRI sites for the introduction of sgRNA-encoding region(s) from pHelper-CRISPRi-sgRNA helper plasmid derivatives (e.g. via restriction/ligation or SLIC cloning).

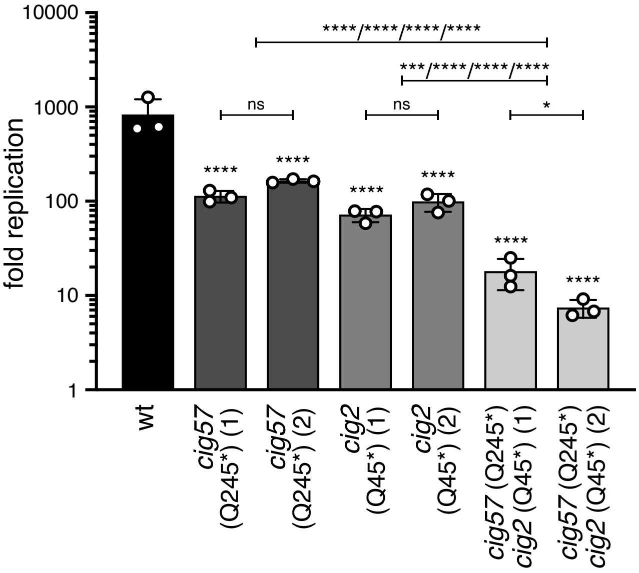

Figure S2 Additional data related to Figure 5. Independently generated clones of base-edited strains display similar phenotypes. Intracellular replication of indicated strains is shown as fold replication (change in genome equivalents (GE) on day 6 post-infection relative to GE on the day of infection). THP-1 cells were infected at an MOI of 0.1. A representative data set is shown. Error bars are SDs. (1) and (2) indicate two independently generated clones harboring the same desired premature stop codon mutation. *$p < 0.05$; ***$p < 0.001$; ****$p < 0.0001$; ns, not significant by one-way ANOVA with Tukey's post hoc test. *p* values shown above bars are in comparison to wild-type; in case of four *p* values displayed above a horizontal line, the order of comparisons is clone (1) vs. clone (1), clone (1) vs. clone (2), clone (2) vs. clone (1), and clone (2) vs. clone (2). wt, wild-type.

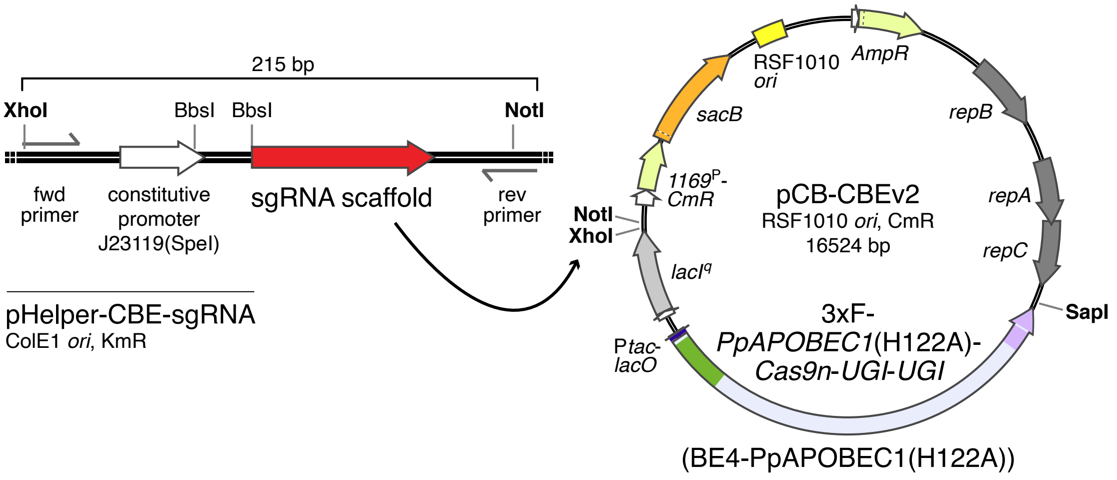

Figure S3 Additional data related to the next-generation *C. burnetii* CBE plasmid (version 2). Plasmid maps of pHelper-CBE-sgRNA (left) and pCB-CBEv2 (right) used for CRISPR-Cas9-mediated cytosine base editing in *C. burnetii* with the improved CBE. pHelper-CBE-sgRNA is a helper plasmid for CBE sgRNA cloning that harbors a sgRNA construct (targeting sequence followed by sgRNA scaffold with Cas9 handle; red) downstream of the synthetic, constitutive bacterial promoter J23119(SpeI) (Registry of Standard Biological Parts). Two BbsI sites allow the insertion of the desired 20 nt targeting sequence as a phosphorylated and annealed oligo duplex. Unique XhoI and NotI sites flank the sgRNA construct for restriction/ligation-mediated subcloning of the sgRNA-encoding region to pCB-CBEv2 (alternatively, the sgRNA-encoding region is moved to pCB-CBEv2 via PCR amplification and e.g. SLIC cloning). pCB-CBEv2 is the next-generation *C. burnetii* CBE plasmid (version 2) without sgRNA construct that encodes the CBE protein 3xF-PpAPOBEC1(H122A)-Cas9n-UGI-UGI (BE4-PpAPOBEC1(H122A)) downstream of the IPTG-inducible P*tac* promoter (IPTG induction is required for base editing in *C. burnetii* using this plasmid). Plasmid contains unique XhoI, NotI, and SapI sites for the introduction of sgRNA-encoding region(s) from pHelper-CBE-sgRNA helper plasmid derivatives (e.g. via restriction/ligation or SLIC cloning). pCB-CBEv2 encodes *sacB*, but *sacB* is not required for base editing protocol. Within the CBE-encoding gene, the cytidine deaminase-encoding sequence is highlighted in green, and the Cas9n- and UGI-encoding sequences are shown in light blue and light violet, respectively.

Supplemental Material Table 1: SNPs identified in strains edited with CBE HF-BE3

***dotA* (Q151*) (SS448)**				
accession nr[†]	*position*	*SNP*	*gene*	*description*
CP020616.1	204027	C→T	*cbu0218a*	pseudogene
CP020616.1	411755	C→T	*cbu0470*	synonymous mutation
CP020616.1	904945	C→T	*cbu0979-cbu0980*	intergenic
CP020616.1	986163	G→A	*cbu1067/ttcA*	nonsynonymous mutation
CP020616.1	1000848	G→A	*cbu1083/mutL*	nonsense mutation
CP020616.1	1349316	G→A	*cbu1427*	pseudogene
CP020616.1	1350314	G→A	*cbu1427*	pseudogene
CP020616.1	1382929	G→A	*cbu1452*	pseudogene
CP020616.1	1507675	C→T	*cbu1588/shaA*	nonsynonymous mutation
CP020616.1	1562505	G→A	*cbu1648/dotA*	desired base edit resulting in Q151*
CP020616.1	1562506	G→A	*cbu1648/dotA*	synonymous mutation, within editing window
CP020616.1	1562616	G→A	*cbu1648/dotA*	synonymous mutation
CP020616.1	1562722	G→A	*cbu1648/dotA*	synonymous mutation
CP020616.1	1562903	G→A	*cbu1648/dotA*	nonsynonymous mutation
CP020616.1	1562916	G→A	*cbu1648/dotA*	nonsynonymous mutation
CP020616.1	1563577	G→A	*cbu1649/icmV-cbu1650/icmW*	intergenic
CP020616.1	1646224	G→A	*cbu1736/purM*	nonsynonymous mutation
CP020616.1	1675172	C→T	*cbu1770*	nonsynonymous mutation
CP020616.1	1698234	C→T	*cbu1790-cbu1792x*	intergenic
CP020616.1	1742936	G→A	*cbu1839*	nonsynonymous mutation
CP020616.1	1962440[‡]	T→A[‡]	*cbu2088*	nonsynonymous mutation
CP020616.1	1964301	C→T	*cbu2090/proC*	synonymous mutation

***cig2* (Q45*) (SS446)**				
accession nr[†]	*position*	*SNP*	*gene*	*description*
CP020616.1	18467	C→T	*cbu0021/cig2*	synonymous mutation, within editing window
CP020616.1	18468	C→T	*cbu0021/cig2*	desired base edit resulting in Q45*
CP020616.1	197076	C→T	*cbu0211*	pseudogene
CP020616.1	209021	C→T	*cbu0226/rplK*	nonsynonymous mutation
CP020616.1	282305	C→T	*cbu0315*	synonymous mutation
CP020616.1	553754	C→T	*cbu0608*	nonsynonymous mutation
CP020616.1	700856	C→T	*cbu0784-cbu0786*	intergenic
CP020616.1	702163	C→T	*cbu0786*	nonsynonymous mutation
CP020616.1	727885	C→T	*cbu0804*	nonsynonymous mutation
CP020616.1	928019	G→A	*cbu1008/bioA*	nonsynonymous mutation
CP020616.1	1083168	G→A	*cbu1164*	pseudogene
CP020616.1	1339694	G→A	*cbu1415/thiL*	nonsynonymous mutation
CP020616.1	1962440[‡]	T→A[‡]	*cbu2088*	nonsynonymous mutation

†: *C. burnetii* Nine Mile RSA439 (phase II, clone 4) reference genome; GenBank accession numbers: CP020616.1 (chromosome) and CP020617.1 (plasmid) (Millar JA *et al.* 2017. *Genome Announc* 5:e00471-17)

‡: Non-CBE-mediated SNP. SNP is also present in parental *C. burnetii* NMII strain (SS364), but with a variant allele frequency (VAF) below threshold used for SNP analysis

Supplemental Material Table 2: SNPs and indels present in all strains sequenced in this study*

accession nr†	*position*	*SNP/indel*	*gene*	*description*
CP020616.1	208721	A→G (E171G)	*cbu0225/nusG*	transcription termination/antitermination protein
CP020616.1	274005	G→A (G111D)	*cbu0307/bbpA*	outer membrane protein
CP020616.1	1479015‡	A→AG‡	*cbu1555*	transposase
CP020617.1	20446‡	T→C‡	*cbuA0020*	hypothetical protein/type IV effector protein?
CP020617.1	20476‡	A→G‡	*cbuA0020*	hypothetical protein/type IV effector protein?
CP020617.1	20495‡	C→T‡	*cbuA0020*	hypothetical protein/type IV effector protein?

*: Including wild-type *C. burnetii* Nine Mile RSA439 (phase II, clone 4) (SS364). Whole genome sequencing results of SNPs and indels identified in all strains sequenced in this study were confirmed by Sanger sequencing

†: *C. burnetii* Nine Mile RSA439 (phase II, clone 4) reference genome; GenBank accession numbers: CP020616.1 (chromosome) and CP020617.1 (plasmid) (Millar JA *et al*. 2017. *Genome Announc* 5:e00471-17)

‡: Possible sequence errors at these 4 positions in *C. burnetii* Nine Mile RSA439 (phase II, clone 4) reference genome; GenBank accession numbers: CP020616.1 (chromosome) and CP020617.1 (plasmid) (Millar JA *et al*. 2017. *Genome Announc* 5:e00471-17). Identified SNPs and indel result in identical alleles as present in *C. burnetii* Nine Mile RSA493 phase I strain; GenBank accession numbers: AE016828.3 (chromosome) and AE016829.2 (plasmid) (Seshadri R *et al*. 2003. *Proc Natl Acad Sci USA* 100:5455-5460)

Supplemental Material Table 3: SNPs identified in strains edited with improved CBE BE4-PpAPOBEC1(H122A)†

***dotA* (Q151*) (48 h IPTG induction; SS504)**				
accession nr‡	*position*	*SNP*	*gene*	*description*
CP020616.1	1562505	G→A	*cbu1648/dotA*	desired base edit resulting in Q151*
CP020616.1	1562510	G→A	*cbu1648/dotA*	nonsynonymous mutation
CP020616.1	1962440§	T→A§	*cbu2088*	nonsynonymous mutation

***cig2* (Q45*) (48 h IPTG induction; SS497)**				
accession nr‡	*position*	*SNP*	*gene*	*description*
CP020616.1	18468	C→T	*cbu0021/cig2*	desired base edit resulting in Q45*
CP020616.1	1403635	G→A	*cbu1475/gatB*	synonymous mutation
CP020616.1	1962871§	C→G§	*cbu2088*	nonsynonymous mutation

***dotA* (Q151*) (extended IPTG induction; SS499)**				
accession nr‡	*position*	*SNP*	*gene*	*description*
CP020616.1	1413326	G→A	*cbu1485-cbu1486*	intergenic
CP020616.1	1541213	G→A	*cbu1627/icmE*	nonsynonymous mutation
CP020616.1	1562505	G→A	*cbu1648/dotA*	desired base edit resulting in Q151*
CP020616.1	1562506	G→A	*cbu1648/dotA*	synonymous mutation, within editing window
CP020616.1	1962871§	C→G§	*cbu2088*	nonsynonymous mutation

***dotA* (Q151*) (extended IPTG induction; SS500)**				
accession nr‡	*position*	*SNP*	*gene*	*description*
CP020616.1	1562505	G→A	*cbu1648/dotA*	desired base edit resulting in Q151*
CP020616.1	1562506	G→A	*cbu1648/dotA*	synonymous mutation, within editing window
CP020616.1	1962440§	T→A§	*cbu2088*	nonsynonymous mutation

***cig2* (Q45*) (extended IPTG induction; SS495)**				
accession nr‡	*position*	*SNP*	*gene*	*description*
CP020616.1	18468	C→T	*cbu0021/cig2*	desired base edit resulting in Q45*
CP020616.1	610174	G→A	*cbu0669*	nonsynonymous mutation
CP020616.1	1866997	C→T	*cbu1981/pdxA*	synonymous mutation
CP020616.1	1962440§	T→A§	*cbu2088*	nonsynonymous mutation

***cig2* (Q45*) (extended IPTG induction; SS496)**				
accession nr‡	*position*	*SNP*	*gene*	*description*
CP020616.1	18468	C→T	*cbu0021/cig2*	desired base edit resulting in Q45*
CP020616.1	20186	C→T	*cbu0021/cig2*	synonymous mutation
CP020616.1	1738915	C→T	*cbu1835*	nonsynonymous mutation
CP020616.1	1755399	G→A	*cbu1853*	nonsynonymous mutation
CP020616.1	1962871§	C→G§	*cbu2088*	nonsynonymous mutation

†: Strains with same desired base edit and identical duration of IPTG induction originate from individual colonies from one transformation with sgRNA-encoding pCB-CBEv2 plasmid derivative

‡: *C. burnetii* Nine Mile RSA439 (phase II, clone 4) reference genome; GenBank accession numbers: CP020616.1 (chromosome) and CP020617.1 (plasmid) (Millar JA *et al.* 2017. *Genome Announc* 5:e00471-17)

§: Non-CBE-mediated SNPs. SNPs are also present in parental *C. burnetii* NMII strain (SS364), but with a variant allele frequency (VAF) below threshold used for SNP analysis
